# Hourglass Mechanism with Temperature Compensation in the Diel Periodicity of Planulation of the Coral, *Seriatopora hystrix*

**DOI:** 10.1371/journal.pone.0064584

**Published:** 2013-05-15

**Authors:** Che-Hung Lin, Keryea Soong, Tung-Yung Fan

**Affiliations:** 1 Institute of Marine Biology, National Sun Yat-Sen University, Kaohsiung, Taiwan, R.O.C.; 2 National Museum of Marine Biology and Aquarium, Pingtung, Taiwan, R.O.C.; 3 Institute of Marine Biodiversity and Evolution, National Dong Hwa University, Pingtung, Taiwan, R.O.C.; University of Texas Southwestern Medical Center, United States of America

## Abstract

Brooding corals often exhibit daily planulation peaks in certain phases of lunar months during their reproductive season. The mechanism controlling this diurnal phenomenon, however, remains uncertain. *Seriatopora hystrix* populations of Southern Taiwan exhibit a highly synchronized planulation rhythm characterized by pre-dawn peak release episodes over a period of 4–6 days per month throughout the year. In this study, controlled light-dark cycles and temperatures were used to study the mechanism of the diel planulation rhythm in the laboratory. *Seriatopora hystrix* did not release planulae under continuous light or continuous darkness. Thus, the lack of free-run did not support a regulatory mechanism involving an endogenous oscillator. Under some lighting conditions, planula release occurred in two peaks per day. Both peaks always occur under dark phases of various light-dark cycles in the laboratory. The first peak occurred when the dark period just started, which might be stimulated by an abrupt transition between light and darkness. The second, pre-dawn peak consistently occurred 23 hrs after application of light. We concluded that this peak of planula release of *S. hystrix* was cued by sunrise of the previous day. Temperature treatments at 1°C intervals from 23.5–28.5°C did not change the diel time of planula release. We suggest that the temperature compensation exhibited in the hourglass model of this species may have a common origin as that of the oscillator model of circadian clocks, due to the similar duration (23 h) and period (∼24 h) between the two. The timing mechanism of planulation discovered here represents an intermediate stage between the hourglass and the oscillator models.

## Introduction

Biological activities often follow a diel pattern [1]. For example, sponges [2], [3], ascidians [4], [5] and polychaetes [6] display diel patterns in different aspects of larvae release, e.g., timing, numbers of peaks and duration of peaks. Spawning of marine organisms such as corals is no exception. Depending on species, gametes or embryos are usually released at certain hours of a day [7], [8], [9], [10], [11]. In some species, a rhythmic pattern could be detected where spawning or planulation occurred in several consecutive days [12].

Two models are often implemented to explain biological rhythms: the endogenous oscillator model and the hourglass model [13]. In the former, clock-like rhythms are autonomous and do not require a cyclic environmental trigger. Pittendrigh [14] summarized that circadian rhythms controlled by a biological clock are characterized by three major features. First, the oscillation can be sustained without external cues (free-running). Second, the rhythmic activities can be entrained by environmental cycles in processes such as light/dark cycles or feeding times (entrainment). Third, despite variations in the surrounding environment, e.g., changes in temperature, an oscillator exhibits little deviation in its period (temperature compensation).

In contrast, in the hourglass model, a rhythm is maintained only because there is oscillation of external environmental cues. The elapsed time between the cue and the expressed activities in the hourglass model corresponds to the reaction time of certain internal processes.

Because temperature controls rates of most chemical reactions, it is often essential in determining the intervals between the environmental cues and the expressed biological activities [15]. Following Van Hoff's Law an increase of 10°C doubles metabolic rates, on average, in a poikilotherm [16], and the estimation of temperature effects could be assessed by:
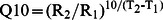
(Q_10_: temperature coefficient, R: metabolic rate, T: temperature).

Hug et al. [17] showed a possibility of hourglass model with temperature compensation theoretically, but so far, no temperature compensation in the hourglass model has been published in living organism, as far as we know.

At least 74 scleractinian coral species are known to be brooders [18]. Most planulation studies focus on annual and lunar reproductive cycles. Planulation is also known to occur in certain hours of a day, most of them at night and before sunrise [11], [12], [19], [20], [21]. The diel timing of planulation may have a significant impact on post-metamorphic success. For example, the settlement rates, metamorphic success and growth rates of pre-dawn planulae were significantly higher than those of post-dusk planulae in the brooding coral, *Favia fragum* [20].

To date, although many studies have described diel times of planulation, which were observed under natural conditions, the mechanisms controlling such rhythmic activities remain little explored. Nevertheless, in *Pocillopora damicornis*, for example, the planulation time was suggested to be controlled by tide [22], [23] and was not affected by light-dark cycles.

*Seriatopora hystrix* is a brooder that releases planulae year round and has a lunar and diel periodicity of planulation: during new moon and 1^st^ quarter moon for 2–3 hours before sunrise in southern Taiwan [11]. The year round reproductive activities provide an opportunity to study the mechanisms. In this research, we aimed to answer the following questions in this work:

1. Is the cyclic planulation of *S. hystrix* directly due to cyclic external light conditions, or, alternatively, an endogenous clock is involved in the rhythm?
2. What is the environmental factor, i.e. sunrise or sunset, determining the diel rhythm of planula release?
3. Does temperature affect the diel timing of planula release?

## Materials and Methods

### Collection sites

All samples in this study were collected with permissions obtained from the Kenting National Park Headquarters. Colonies of *S. hystrix* were collected at 3–10 m depth from two fringing reefs of Southern Taiwan, Leidashyr (21°55′89″N, 120°45′69″E) and Hobihu (21°56′29″N, 120°44′70″E). Colonies collected in whole were harvested using a hammer and a chisel before new moons, with maximum diameters ranged from 7–23 cm. The average colony size was 14.7±5.4 cm (mean ± S.D, n = 50), and all corals were kept in the culturing facility in National Museum of Marine Biology and Aquarium. Each colony was placed in a separate flow-through tank, (22×15×18 cm L × W × H) in dimensions, with an overflowing outlet of 2 cm in diameter. The source seawater was pumped from a 5-m depth, filtered to 50 µm, and injected into the bottom of the tanks. The outflow of each tank passed through a collecting cup with a sieve made from a plankton net (mesh size 100 μm) to trap the released planulae. The planulae collected in each cup were counted hourly, during the experiments.

Tanks were placed in a dark room with light controls. Each treatment equipped with two OSRAM HQI-TS 70W/D metal halide lamps (photosynthetically active radiation (PAR) ∼150μE m^−^^2^ s^−^^1^) was subjected to different light regimes.

### Free-run experiment

Twelve adult colonies of *S. hystrix* were collected in February, 2006 and were kept under the condition of LD (light-dark) cycle 11∶13 (light-on at 0700 hr). The LD cycle was designed in order to simulate that in the field. Planulation occurred twelve days later. After the start of planulation, four colonies were used in each of the three treatments. In the control groups, the light regime was maintained at LD 11∶13 throughout the experiment. In the LL (continual light) treatment groups, the application of continuous light sustained for 90 hours then changed to LD 11∶13. In the DD (continual dark) treatment groups, continuous darkness lasted for 97 hours, after which colonies were exposed to LD 11∶13. Data from each colony of the same treatments were pooled.

### Entrainment experiment

Twenty adult colonies were collected and maintained at LD 12∶12 (light-on at 0700 hr, light-off at 1900 hr) for nine days until the start of planulation in January, 2007. Then, four colonies were used in each of the five experimental treatments. In the control groups, an LD 12∶12 was used, with light-on at 0700 hr. To examine the effect of light-on time, an LD 19∶5, with light-on at 0000 hr and light-off at 1900 hr was used in group A, and an LD 5∶19, with light-on at 1400 hr and light-off at 1900 hr was used in group B, respectively. To examine the effect of light-off time, an LD 19∶5, with light-off at 0200 hr and light-on at 0700 hr was used in group C, and an LD 5∶19, with light-off at 1200 hr and light-on at 0700 hr was used in group D, The numbers of planulae released by each colony were recorded hourly for five consecutive days.

### Temperature compensation experiment

Eighteen adult colonies were collected and maintained at LD 12∶12 in March, 2007. After the colonies started releasing planulae, they were randomly divided into 6 groups. Each group was assigned to one of the water temperature treatments at 23.5, 24.5, 25.5, 26.5, 27.5 and 28.5°C. Three colonies, in separate flow-through tanks, were used in each temperature treatment. In order to manipulate inflow seawater to the corresponding designated temperatures, six 130-liter PVC tanks equipped with heaters and chillers were used. Temperature-manipulated water then was pumped to individual flow-through tanks in each treatment, respectively. HOBO Temperature Data Loggers (Onset Computer Corp., Bourne, MA) were used to record the temperature every 5 minutes in the treatment tanks to keep track of the experimental conditions. The numbers of planulae released were recorded every hour for three consecutive days. Data from each colony were transformed to percentages each day. The average times of planula release in each temperature treatment were used to calculate Q_10_, using the formula mentioned prevously.

The data of bleached colonies or those releasing fewer than 10 planulae per day per colony were excluded from the analyses.

## Results

### Free-run experiment

**Figure 1:**
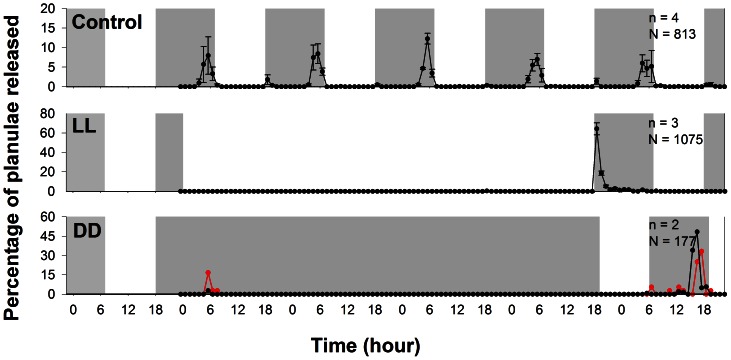
The distribution of planula release in the free-run experiment. Data are expressed as mean ± S.E., n =  number of coral colonies in each treatment, N =  total number of planulae in each treatment. Note that only two colonies in the DD treatment released planulae during the experiment. The gray and white areas represent the dark and light phases, respectively.

In the control group with LD 11∶13, the rhythmic planulation peak occurred around 0500, i.e., 22.9±0.14 hrs (mean ± S.E.) after the onset of light, every day. A few planulae (fewer than 20 planulae/colony) were released 1–2 hours after the start of light-off (Fig. 1). In LL group, no planula release was observed for 90 consecutive hours until light-off, when a high peak of planulation occurred; there was no pre-dawn peak of planulation (Fig. 1). In DD, a peak occurred at 0500 on the first day, then no planulae were released for 96 h of continuous darkness. A peak of planulation was observed toward the end of the dark phase, after LD cycle resumed (Fig. 1). Free-running was not observed in this experiment.

### Entrainment experiment

**Figure 2:**
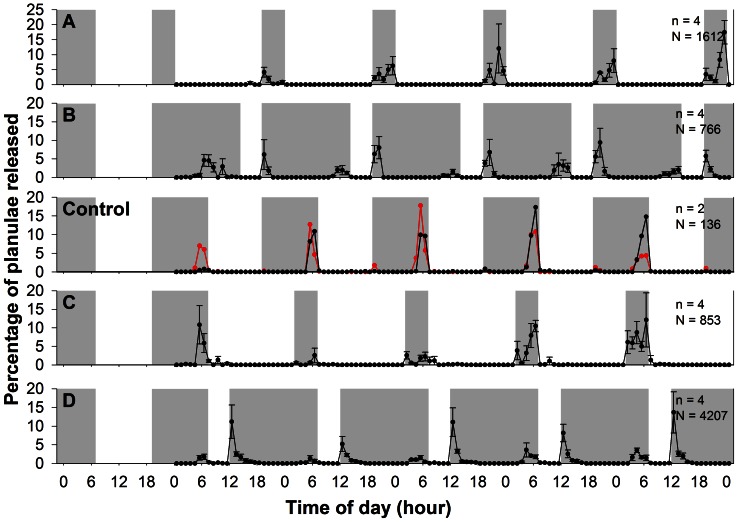
The effect of light regime on planulae release in the entrainment experiment. Data are expressed as mean ± S.E., n =  coral colonies in each treatment, N =  total number of planulae in each treatment. Note that only two colonies in the control group released planulae during the experiment. The gray and white areas represent the dark and light phases, respectively.

In this experiment, daily synchronous planulation occurred in two main peaks, the first peaks occurred after light-off and second peaks occurred before light-on during the experimental period (Fig. 2).

The second peak of group A was about 7 hours earlier than the control group; the second peak of group B was about 7 hours later than that in the control group; the second peak of groups C and D were similar to those of the control group (Fig. 2).

**Figure 3:**
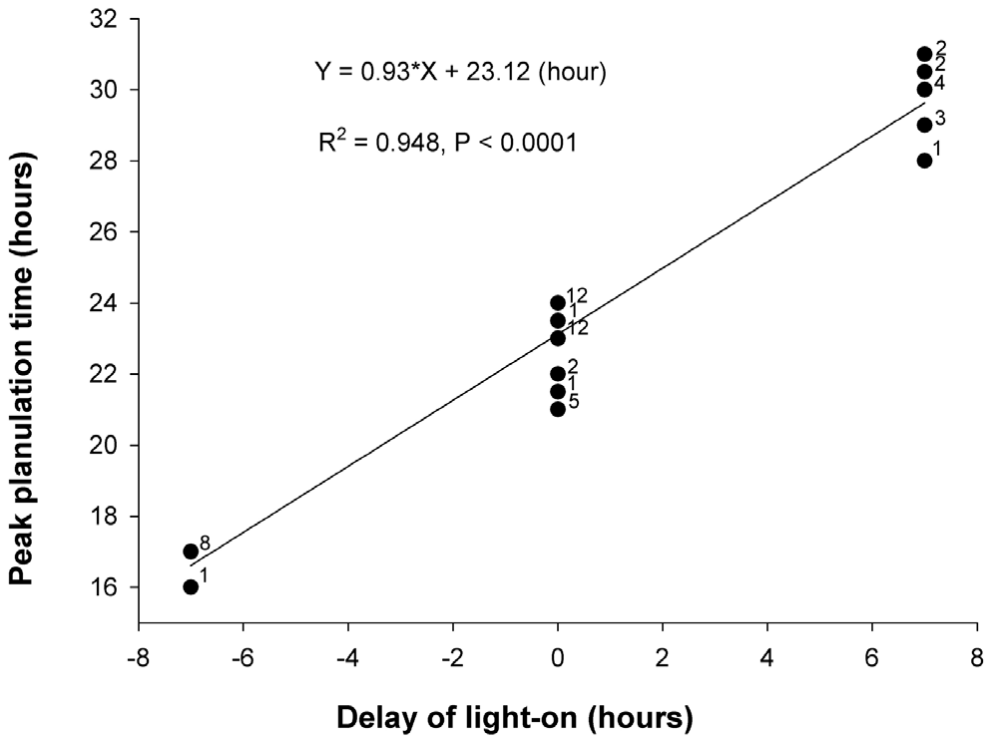
Regression analysis between planulation time and the delay of light-on in the entrainment experiment. Numbers next to solid dots indicate numbers of observations. Data from Figure 2. Each dot represents data from one colony on each day.

The intervals between light-on and the second peak of planulation were about 23 hours in the control group. In comparing treatments, a delay in hours of light-on (X) resulted in a corresponding delay in the second peak of planulation (Y) (Y = 0.93X+23.12 (h), P<0.01, R^2^ = 0.95, 99% C.I. of slope  = 0.08, Fig. 3). Thus, the intervals between lights-on and the second peaks of planulation were approximately 23 hours, despite various photoperiods.

On the other hand, there was no relationship between the second peak of planulation and the time of light-off (P = 0.31, R^2^ = 0.02). This result indicated the role of light-on, but not light-off, on the diel periodicity of the second peak of planulation.

### Temperature compensation experiment

**Figure 4:**
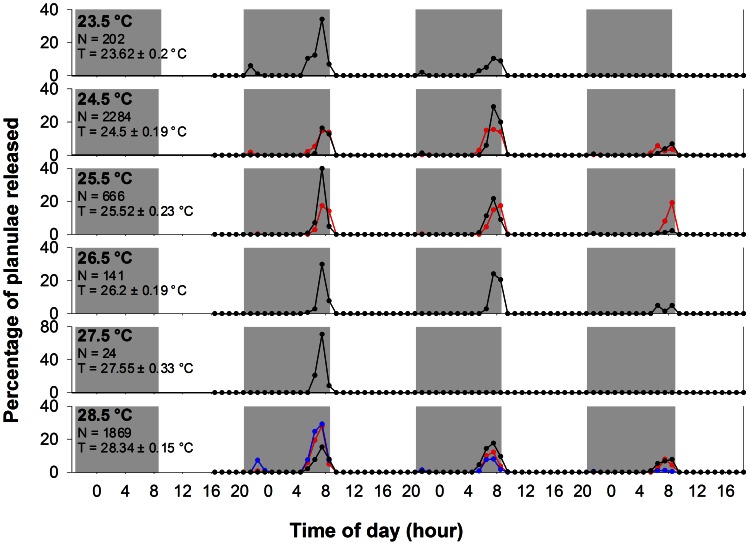
Planulae release time under different temperature treatments. Data from different colonies are shown in separate lines. N =  total number of planulae in each treatment, T =  actual temperature in each treatment (mean ± S.D.). There were no significant correlations between elapsed time (interval between light-on and the planulation peak) and temperature (P = 0.97, Q_10_ = 1.01).

All of the planulation events occurred during the dark period in this experiment. The timing of the main planulation peaks was similar among treatments, that is, 23.15±0.09 hrs (Fig. 4) after application of light. The mean water temperature during the experimental period received by each treatment tank respectively were 23.62±0.2, 24.5±0.19, 25.52±0.23, 26.2±0.19, 27.55±0.33 and 28.34±0.15°C (23.5, 24.5, 25.5, 26.5, 27.5 and F treatment, mean ± SD). A regression analysis revealed no significant relationship between peaks of planulation time (Y) and water temperatures (X) (Y = −0.0017X +23.2 (h), P = 0.97); moreover, the calculated Q_10_ of 1.01 was not significantly different from 1.

## Discussion

In this study, we found that *S. hystrix* colonies did not release planulae during continuous light and continuous dark conditions (Fig. 1). This result suggests the absence of free-runing which is the property of a circadian oscillator. Furthermore, the timing of planulae release corresponded with shifting in timing of light-on on the days of the treatments (Figs. 2 and 3). Thus, the hourglass model apply here with regard to the timing of larval release. It appears, on the other hand, that the onset of light phase is the trigger that initiates the processes leading to planulation in *S. hystrix*. Therefore, we surmise that the rhythmic planulation of *S. hystrix* is due to the cyclic sunrise in nature. Interestingly, the oscillator model of coral spawning was also rejected in a study of the broadcasting coral, *Montastraea franksi*, in which spawn timing was directly controlled by sunset time [24].

In this study, the observed pattern of the two-peak planulation strategy differed from the results of a previous study [11] in which only one planulation peak (pre-dawn) occurred in a day. We suggest that an abrupt transition from light to dark may have a role in the occurrence of these planulation peaks. In our experiment, “rectangular” light/dark stimulations (i.e., abrupt transition from light to dark and vice versa) was employed. On the other hand, Fan et al. [11] used natural light, which includes twilight and dusk periods (i.e., gradual transitions from light to dark and vice versa). Similar results were also observed in mammals. Specifically, it is evident that twilight periods increase the strength of the LD synchronizer, as compared with studies using abrupt LD transitions, in squirrel monkeys [25] and hamsters [26], [27]. However, similar activity patterns were observed in birds exposed to these two regimes [28].

The phenomenon of two-peak planulation under natural light condition was reported in *P. damicornis* and *Stylophora pistillata, in the same family as S. hystrix*. In *P. damicornis*, planulation occurred throughout the day with one major pre-dawn peak and one minor post-dusk peak [11]. Planulation of *S. pistillata* was shown to have one pre-dawn peak, but sometimes, a slight peak occurred after sunset (see figure 3 of [11]). The two-peak planulation strategy in these three species of relatively recent common ancestry suggests that the minor post-dusk peak could be a vestigial trait. This hypothesis is supported by the phenomenon that the post-dusk planulation peaks occurred only under certain artificial light condition in *S. hystrix*.

The two planulation peaks demonstrated by *S. hystrix* within the dark phase of one day brings to mind the two locomotor activity peaks in *Drosophila pseudoobscura* [29]. In this fruit fly, the appearance of the first peak of activity after lights were turned on was due to a masking effect, while the second peak (before light-off) was triggered by an oscillator. In *S. hystrix*, the post-dusk peaks and the pre-dawn peaks may be regulated by the masking effect and the hourglass model, respectively. In this case, the post-dusk peak of planulation could also be an hourglass mechanism with very short duration between dusk and planulation. On the other hand, the pre-dawn peaks could not be controlled by a masking effect which dictates an immediate response after the cues. The possible independence of the two peaks is supported by the wide ranges of the intervals between them, which were from 2 to 16 hours. On the other hand, Aschoff & von Goetz (1989) hypothesized that the degree of the masking effect was dependent on circadian phases; under LD 4∶20, the degree of masking effect was higher than under that under LD 12∶12. This hypothesis is supported in our study, as the number of planulae released of post-dusk peak in LD 5∶19 treatment was higher than that those of the LD 19∶5 and LD 12∶12 treatments (Fig 2).

Rensing et al. [13] suggested that the timer in the hourglass model is supposed to be the consumption of a critical substrate in a rate determining step. Therefore, the Van't Hoff rule, i.e. reaction rates double for every 10°C rise in temperature, should apply. Concerning the hourglass model and Van't Hoff rule, temperature compensation should not occur in hourglass model of timing mechanisms. This is obviously not supported by our study, which found that the temperature did not affect the peak larval release times.

In this study, we noticed that the interval between the cue and the planulation of *S. hystrix* is 23.2 hrs, very close to the periods of circadian oscillators. This finding prompt us to consider the origin of the two mechanisms. It could be a result of convergent evolution due to strong selective pressures in corals to planulate at an exact hour. On the other hand, the existence of temperature compensation, a property of the oscillator model, suggests that there is a common trait between the two rhythm models. That hourglass clock may be a special oscillator with fast damping of the cyclic activities was hypothesized in prior studies [30], [31], [32]. The results here suggest that the mechanism of planulation timing in *S. hystrix* could be an intermediate stage between the typical hourglass and the oscillator models.

Two hypotheses have been proposed to explain the adaptive values of diel timing of planulation. (1) Predator avoidance: planulae released at night may have a high survival rate since it is easy to avoid predators that rely on vision for foraging [9], [33], [34], [35]. (2) Reducing duration of the swimming stage of planulae: settlement of planulae may require visual cues such as light orientation. Planulae released pre-dawn may be able to find suitable substrate with minimal waiting. Goodbody-Gringley (2010) observed that *Favia fragum* planulae released pre-dawn had significantly higher rates of settlement than those released post-dusk. The latter hypothesis, in particular, could explain the propensity for a pre-dawn release in *S. hystrix* and other reef-building corals. For those corals of Taiwan in particular, it also seems likely that light, as opposed to temperature, is a larger driver of larval release timing because of the thermodynamically unstable nature of the reefs of Southern Taiwan [36], in which the local water temperature may fluctuate 10°C within 24 h due to tidally induced upwelling [36]. Additionally, the monthly average temperature differs by about 5°C in a year. Without a mechanism for temperature compensation, the time of planulation could deviate by several hours from sunrise.

Our conclusion of the mechanism of planulation rhythm of *S. hystrix* is that it is under regulation of a hourglass model with an involvement of temperature compensation.
